# Supplementary material for: A Critical View of the Application of the APEX Software (Aqueous Photochemistry of Environmentally-Occurring Xenobiotics) to Predict Photoreaction Kinetics in Surface Freshwaters
Source: Molecules. 2019 Dec 18;25(1):9. doi: 10.3390/molecules25010009 (PMC7017383; doi:10.3390/molecules25010009)
Supplement: Supplementary file 1 [file molecules-25-00009-s001.zip › 3.2.4_gcc-4.4.0.zip!/3.2.4_gcc-4.4.0/3.2.4_gcc-4.4.0/doc/octave/html/interpreter/Advanced-Plotting.html]

Advanced Plotting - Untitled


Previous: Plotting Basics,
Up: Plotting


---

### 15.2 Advanced Plotting

- Graphics Objects- Graphics Object Properties- Managing Default Properties- Colors- Line Styles- Marker Styles- Callbacks- Object Groups- Graphics backends
